# Cholecystokinin octapeptide improves hippocampal glutamatergic synaptogenesis and postoperative cognition by inhibiting induction of A1 reactive astrocytes in aged mice

**DOI:** 10.1111/cns.13718

**Published:** 2021-08-17

**Authors:** Lei Chen, Ning Yang, Yue Li, Yitong Li, Jingshu Hong, Qian Wang, Kaixi Liu, Dengyang Han, Yongzheng Han, Xinning Mi, Chengmei Shi, Ying Zhou, Zhengqian Li, Taotao Liu, Xiangyang Guo

**Affiliations:** ^1^ Department of Anesthesiology Peking University Third Hospital Beijing China; ^2^ Department of Anesthesiology Plastic Surgery Hospital Chinese Academy of Medical Sciences and Peking Union Medical College Beijing China

**Keywords:** A1 reactive astrocyte, activated microglia, cholecystokinin octapeptide, delayed neurocognitive recovery, glutamatergic synaptogenesis

## Abstract

**Aims:**

Delayed neurocognitive recovery (dNCR) is a common postoperative complication in geriatric surgical patients for which there is no efficacious therapy. Cholecystokinin octapeptide (CCK‐8), an immunomodulatory peptide, regulates memory and learning. Here, we explored the effects and mechanism of action of CCK‐8 on dNCR.

**Methods:**

We applied laparotomy to establish a model of dNCR in aged mice. Morris water maze and fear conditioning tests were used to evaluate cognition. Immunofluorescence was used to detect the density of CCK‐8, A1 reactive astrocytes, glutamatergic synapses, and activation of microglia in the hippocampus. Quantitative PCR was performed to determine mRNA levels of synapse‐associated factors. A1 reactive astrocytes, activated microglia, and glutamatergic synapse‐associated protein levels in the hippocampus were assessed by western blotting.

**Results:**

Administration of CCK‐8 suppressed the activation of microglia, the induction of A1 reactive astrocytes, and the expression of tumor necrosis factor alpha, complement 1q, and interleukin 1 alpha in the hippocampus. Furthermore, it promoted glutamatergic synaptogenesis and neurocognitive recovery in aged dNCR model mice.

**Conclusion:**

Our findings indicated that CCK‐8 alleviated cognitive impairment and promoted glutamatergic synaptogenesis by inhibiting the induction of A1 reactive astrocytes and the activation of microglia. CCK‐8 is, therefore, a potential therapeutic target for dNCR.

## Graphical abstract

Cholecystokinin octapeptide (CCK‐8) alleviated cognitive impairment and promoted glutamatergic synaptogenesis by inhibiting the induction of A1 reactive astrocytes, which was induced by activated microglia‐released tumor necrosis factor alpha (TNF‐α), complement component 1, q subcomponent (C1q) and interleukin 1 alpha (IL‐1α). These findings demonstrated that CCK‐8 is a potential therapeutic target for delayed neurocognitive recovery (dNCR).

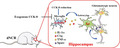

## 1 INTRODUCTION

Delayed neurocognitive recovery (dNCR) is a common postoperative complication in elderly surgical patients,^1^ which is defined as cognitive decline up to 30 days postoperatively and characterized by impaired memory, learning, and attention.^2^ dNCR is associated with longer hospital stays, higher mortality and heavier social burden.^3^ However, the neuropathogenesis of dNCR is unclear and lack of effective treatment.

Astrocytes, as the most abundant glial cell in the central nervous system (CNS), play a key role in health and disease.^4^ Extensively branched processes of astrocytes are in close proximity to synapses; therefore, their role in regulating the functions of synapses has been widely discussed.^5^, ^6^, ^7^ Different properties of activated astrocytes have been demonstrated in age‐associated CNS diseases, such as Alzheimer's (AD), Parkinson's (PD), and Huntington's (HD) diseases.^8^, ^9^ Reactive astrocytes are classified as neurotoxic type A1 and neuroprotective type A2. Numbers of A1 reactive astrocytes are increased in the brain of AD, PD, HD, and multiple sclerosis patients.^10^ Several studies show that A1 reactive astrocytes are induced by activated microglia‐released factors, including tumor necrosis factor alpha (TNF‐α), complement component 1 q subcomponent (C1q) and interleukin 1 alpha (IL‐1α).^10^, ^11^ Fei et al.^12^ demonstrated that etomidate (a common clinical general anesthetic)‐induced long‐term synaptic inhibition and cognitive dysfunction via A1 reactive astrocytes in aged mice. However, it is still unknown that whether the A1 reactive astrocytes participates in the dNCR.

Astrocyte‐secreted neurotransmitters, such as glutamate and D‐serine, modulate the functions of synapses. Additionally, astrocyte‐secreted factors, such as thrombospondins (THBS), secreted protein acidic and rich in cysteine (SPARC)‐like 1, and glypican (GPC), promote synaptogenesis.^13^ Meanwhile, decreased hippocampal glutamatergic synapse density is associated with cognitive dysfunction.^14^ Whereas, synaptogenesis‐associated factors secreted by astrocyte (THBS, SPARCL1 and GPC) has not been reported in dNCR. Therefore, in the present study, we investigated the neurotoxicity of A1 reactive astrocytes on hippocampal glutamatergic synaptogenesis and the changes of synaptogenesis‐associated factors in dNCR.

Cholecystokinin (CCK) is a 33‐amino acid peptide hormone found in the gastrointestinal tract and it is the most abundant peptide neurotransmitter in the brain,^15^ with especially high levels in the hippocampus, amygdala, hypothalamus, and ventral tegmental area.^16^ CCK regulates feeding, learning, memory, and nociception by binding to two G‐protein receptors: CCK1 and CCK2.^17^, ^18^, ^19^ CCK has numerous isoforms, including CCK‐58, CCK‐22, CCK‐8, CCK‐5, and CCK‐4.^20^ CCK‐8 is one of the most abundant isoforms and mediates the biological functions of CCK in the CNS.^21^ Although several studies have confirmed the involvement of CCK in aging and neurodegenerative disease‐induced memory impairment,^22^, ^23^, ^24^ the functions of CCK in perioperative cognitive impairment have not yet been reported.

In the present study, we found that surgery/anesthesia decreased the levels of CCK‐8 in the hippocampus, especially in CA1 and dentate gyrus (DG) regions. Asrican et al. showed that reduced CCK abundance induced reactive astrocytes and increased the expression of genes involved in neuroinflammation in the DG.^25^ Therefore, we speculated that induction of A1 reactive astrocytes by reduced levels of CCK‐8 participates in the neuropathogenesis of dNCR. Furthermore, we explored the cognitive protective effect and possible mechanism of action of CCK‐8.

## 2 MATERIALS AND METHODS

### 2.1 Animals

**FIGURE 1:**
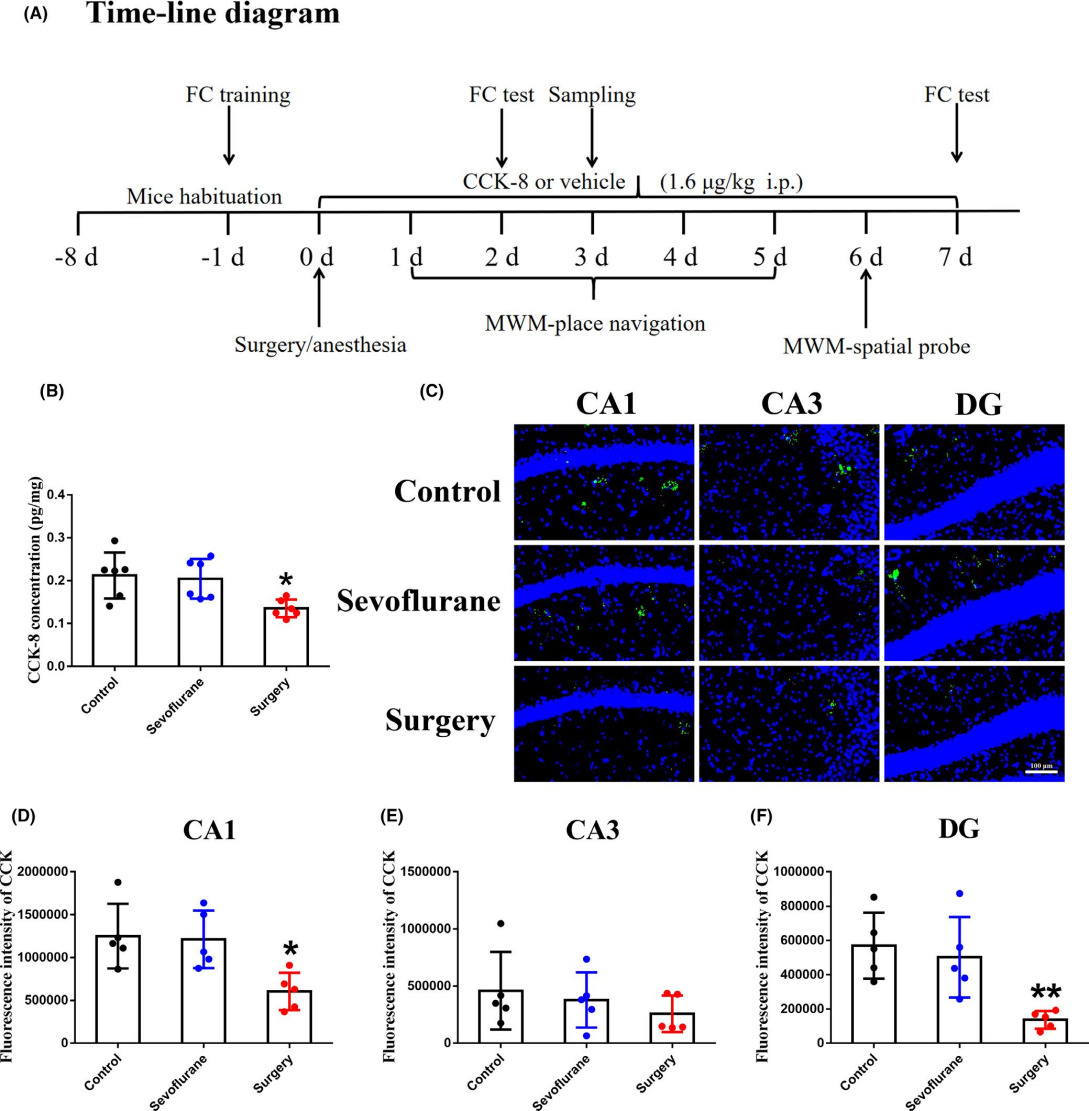
Surgery/anesthesia decreases the levels of CCK‐8 in the hippocampus of aged mice. (A) Timeline of the experiment. (B) ELISA detection of CCK‐8 levels in the hippocampus 3 days after surgery (*n* = 6 per group). (C) Immunofluorescence detection of CCK‐8 levels in CA1 (C, D), CA3 (C, E), and DG (C, F) regions 3 days after surgery (*n* = 5 per group). Data are expressed as the mean ±SEM (one‐way ANOVA followed by Bonferroni's post hoc test).*p < 0.05, **p < 0.01 versus control and sevoflurane groups. FC, fear conditioning; CCK‐8, cholecystokinin octapeptide; MWM, Morris Water Maze

Aged female C57BL/6 mice (18 months old, 30–40 g) were purchased from Tianqin Biotechnology Company Limited (Changsha, China). The mice were group housed, five per cage, with a 12 h light/dark cycle in a temperature‐controlled (24 ± 1℃) room with free access to food and water in accordance with the standards established by the Experimental Animal Laboratory. Mice were acclimatized to these housing conditions for a week before commencement of the experiment. Every effort was made to minimize the pain and discomfort of animals. The study protocol was approved by the Animal Ethics Committee of Peking University Health Science Center (LA2021423) and conducted in accordance with the Guiding Principles for the Care and Use of Animals in Research, the ARRIVE 2.0 guidelines.^26^ The diagram of the experiment is shown in Figure 1A.

### 2.2 dNCR model

The dNCR mouse model was established in accordance with previous studies.^27^, ^28^, ^29^ Briefly, mice were anesthetized with 5% sevoflurane for 3 min and maintained with 2.0%–2.5% sevoflurane in 40% oxygen. After the surgical site was shaved and sterilized, a 1.5 cm vertical incision was made 0.5 cm below the right costal margin through the skin and muscle wall. After exploring the abdominal organs (liver, spleen, kidneys, and bowel), a 10 cm section of the small intestine was removed from the abdominal cavity and rubbed vigorously between the index finger and thumb for 30 s. The small intestine was placed back into the abdominal cavity and the incision closed with 4–0 sutures. Sevoflurane anesthesia was stopped and lidocaine cream was applied to reduce incision pain every eight hours for three days after surgery. During this procedure, which lasted ~30 min, mice were placed on a heating pad to maintain body temperature. Mice in the sevoflurane group received the same sevoflurane exposure without laparotomy, while mice in the control group received no treatment.

### 2.3 Drug administration

Cholecystokinin octapeptide (CCK‐8) was purchased from Tocris Bioscience Ltd (Tocris Cookson, Northpoint, UK, 1166) and dissolved in phosphate‐buffered saline (PBS, pH 7.4). According to previous reports,^15^, ^30^ CCK‐8 was thawed daily and administered intraperitoneally at a dose of 1.6 μg/kg. The control and surgery groups received an equivalent volume of PBS vehicle (2 ml/kg).

### 2.4 Morris water maze

The Morris water maze (MWM) is used for hippocampus‐dependent tests that reflect the spatial navigation and reference memory of rodents.^31^ The MWM (Sunny Instruments Co. Ltd., Beijing, China) consists of a circular white tank (120 cm in diameter and 50 cm high) containing water (23 ± 1℃) that is divided into four quadrants and a platform (10 cm in diameter) located 1 cm below the water in the target quadrant. In the place navigation test, the mice were placed in one quadrant facing the wall of the maze and allowed to explore for the hidden platform for 90 s in each trial (four trials per day with an intertrial interval of 5 min). The time to locate the submerged platform was recorded (defined as the escape latency). If the platform was not found within 90 s, the mice were guided to the platform, where they stayed for 15 s. Mice underwent daily testing in the water maze from day 1 to day 5 after surgery. On postoperative day 6, the submerged platform was removed from the water maze and a spatial probe test was performed for 60 s. The swimming speed, escape latency, number of platform crossings, and the time spent in the target quadrant were recorded by a video camera.

### 2.5 Fear conditioning

Fear conditioning consists of a training phase to establish long‐term memory and a testing phase.^32^, ^33^, ^34^ Training was performed 1 day prior to surgery. Mice were allowed to adapt to the conditioning chamber for 2 min, and then a 70 dB tone was played (conditional stimulus, 20 s) followed by a trace interval of 25 s. Next, a 2 s 0.7 mA electric foot‐shock was administered (unconditional stimulus). Six pairs of conditional‐unconditional stimuli with 60 s intervals between each pair were administered. The mice were then allowed to stay in the chamber for 60 s.

The testing phase consisted of a context test and a tone test. In the context test, mice were put back in the chamber for 5 min without tone or electric foot‐shock. The tone test was performed 2 h after the context test. Mice were placed in a chamber that was different from the training phase for 5 min. During this period, mice were given sound stimuli (70 dB 3 min) without electric foot‐shock. The total distance and freezing time were recorded using tracking system software (Macroambition S&T Development Co. Ltd., Beijing, China). After each test session, the chamber was wiped with 75% alcohol to avoid an odor effect.

### 2.6 Western blotting

Hippocampus samples were homogenized using radioimmunoprecipitation lysis buffer containing protease and phosphatase inhibitors. The lysate was centrifuged at 15,000 g for 10 min at 4℃ to remove debris. Supernatants were collected, and protein concentration was determined using a bicinchoninic acid protein assay kit (Applygen, Beijing, China, P1511). Protein samples (40 μg) were separated by 10% sodium dodecyl‐sulfate polyacrylamide gel electrophoresis and then transferred to polyvinylidene difluoride membranes (Millipore, Billerica, MA, IPVH00010). After blocking with 5% Albumin Bovine V (Solarbio, Beijing, China, A8020) in TBST (Tris‐buffered saline containing 0.1% Tween 20) for 1 h at room temperature, membranes were incubated overnight at 4℃ with the following primary antibodies: anti‐IL‐1α (1:1000, Santa Cruz Biotechnology, sc‐12741), anti‐TNF‐α (1:1000, Santa Cruz Biotechnology, sc‐52746), anti‐C1q (1:1000, Santa Cruz Biotechnology, sc‐365301), anti‐postsynaptic density (PSD)95 (1:1000, Cell Signaling Technology, 36233), anti‐vesicular glutamate transporter (vGLUT)1 (1:1000, Abcam, ab227805), anti‐GFAP (1:1000, Cell Signaling Technology, 3670), anti‐complement component 3 (C3) (1:1000, Proteintech, 21337–1‐AP), or anti‐β‐actin (1:1000, Santa Cruz Biotechnology, sc‐47778). Membranes were washed three times with TBST and incubated with horseradish peroxidase‐conjugated secondary antibody for 1 hour at room temperature. The membranes were then exposed to a chemiluminescence reagent (TianGen, Beijing, China, PA112‐02) and then analyzed using Image J (National Institutes of Health, USA).

### 2.7 Immunofluorescence

Mice were deeply anesthetized with an overdose of sevoflurane and transcardially perfused with PBS, followed by 4% paraformaldehyde in PBS. Brains were removed and post‐fixed in 4% paraformaldehyde at 4℃ for 24 h. Brains were then coronally sectioned at 30 µm using a cryostat microtome (Leica, Weztlar, Germany, CM3050S,). Free‐floating sections were washed with PBS (three times, 8 min each), rinsed and then transferred to blocking buffer (10% normal goat serum, 0.3% Triton X‐100 in PBS) for 1 h at 37℃. After blocking, the sections were incubated with mouse anti‐PSD95 (1:100, Cell Signaling Technology, 36233) and rabbit anti‐vGLUT1 (1:100, Abcam, ab227805). After washing in PBS (3 times, 10 min each), the slices were incubated with FITC‐labeled goat anti‐rabbit (1:200, ZSGB‐Bio, Beijing, China, ZF‐0311) and TRITC‐labeled goat anti‐mouse (1:200, ZSGB‐Bio, ZF‐0313) secondary antibodies for 2 h at 37℃, stained with DAPI for 10 min at room temperature (25 ± 2℃), washed with PBS (3 times, 10 min each), and mounted in 70% glycerol. Sections were also incubated with rabbit anti‐IBA1 (1:500, Wako, 019‐19741), mouse anti‐GFAP (1:100, Cell Signaling Technology, 3670) and rabbit anti‐C3 (1:50, Proteintech, 21337‐1‐AP) as above. Finally, the immunostained sections were imaged using a TCS SP8 X confocal fluorescence microscope (Mannheim, Germany) or a virtual microscopy slide‐scanning system (VS 120, Olympus, Japan). Images of sections containing the target brain regions were cropped and analyzed using ImageJ (National Institutes of Health).

### 2.8 Quantitative real‐time PCR (qPCR)

Total RNA was extracted from the hippocampus using TRIzol (TianGen, Beijing, China, DP424) in accordance with the manufacturer's protocol. Total RNA concentration and purity were determined using a NanoDrop spectrophotometer (NanoDrop Technologies). RNA samples were reverse transcribed into cDNA using the Fastking 1st Strand cDNA Synthesis Kit (TianGen, KR116) and qPCR was performed using SYBR Green Talent qPCR PreMix (TianGen, FP209) according to the manufacturer's instructions. β‐actin was used as an internal control. Relative gene expression was calculated using the 2^−ΔΔCt^ method and the primers used are listed in Table 1.

**TABLE 1:** Primers Sequence for qPCR

ID	FORWARD	REVERSE
*Gpc4*	TTGACACCAGCAAGCCAGACATAC	ATCCGCTTCCACTTCCCTCTCC
*Gpc6*	AACGGACACAGCAAAGCCAGATAC	TTTGTTGGTCATCACACGGAGAGC
*Sparc*	TGTCCTGGTCACCTTGTACGAGAG	TGGATCTTCTTCACACGCAGCTTC
*Sparcl1*	CACATACAGAGCAGCAGGACCAAG	CACGGCAGCATCGGTAGGTTC
*Thbs1*	ATGCCTGCGATGATGACGATGAC	CTGGGCTGGGTTGTAATGGAATGG
*Thbs2*	AGACCAGGAAGACTCGGACG	TGGCATTGTTCTCAGGGCAC
*β‐actin*	GTACCACCATGTACCCAGGC	AACGCAGCTCAGTAACAGTCC

### 2.9 Enzyme‐linked immunosorbent assay (ELISA)

The concentration of CCK‐8 in the hippocampus was quantified using an ELISA kit in accordance with the manufacturer's instructions (Reddot Biotech, British Columbia, Canada, RD‐CCK‐8‐Mu).

### 2.10 Statistical analysis

GraphPad Prism 6 (GraphPad, New York, USA) was used for statistical analyses. The Shapiro‐Wilk test was used to analyze the normality of the data, and we found that the data were normally distributed. Quantitative data are expressed as the mean ± standard error of the mean (SEM). Statistical significance was determined using analysis of variance (ANOVA), followed by Bonferroni's post hoc test. A *p*‐value <0.05 was considered statistically significant. Statistical power analysis was used to verify significant differences with respect to sample size (GPower 3, ≥0.8 for sufficient power validation).^35^

## 3 RESULTS

### 3.1 Surgery/anesthesia decreases the levels of CCK‐8 in the hippocampus of aged mice

We previously showed that 30 min of inhalation anesthesia alone did not induce cognitive impairment in mice.^32^, ^36^ Here, we examined the effect of sevoflurane anesthesia with or without surgical trauma on the levels of CCK‐8 in the hippocampus of aged mice. We found no significant differences in CCK‐8 levels between the control and sevoflurane groups (Figure 1B–F), indicating that sevoflurane anesthesia alone did not change the levels of CCK‐8 in the hippocampus of aged mice. However, surgery/anesthesia significantly decreased the levels of CCK‐8 in the hippocampus (Figure 1B) of aged mice, especially in the CA1 (Figure 1C, D) and DG regions (Figure 1C, F). These findings indicated that surgery/anesthesia‐induced cognitive impairment in aged mice may be associated with reduced levels of CCK‐8 in the hippocampus.

### 3.2 CCK‐8 alleviates hippocampus‐dependent memory impairment in aged dNCR model mice

On the account of the postoperative reduction in CCK‐8 levels, we explored the effect of CCK‐8 supplementation on cognitive function in aged dNCR model mice.

**FIGURE 2:**
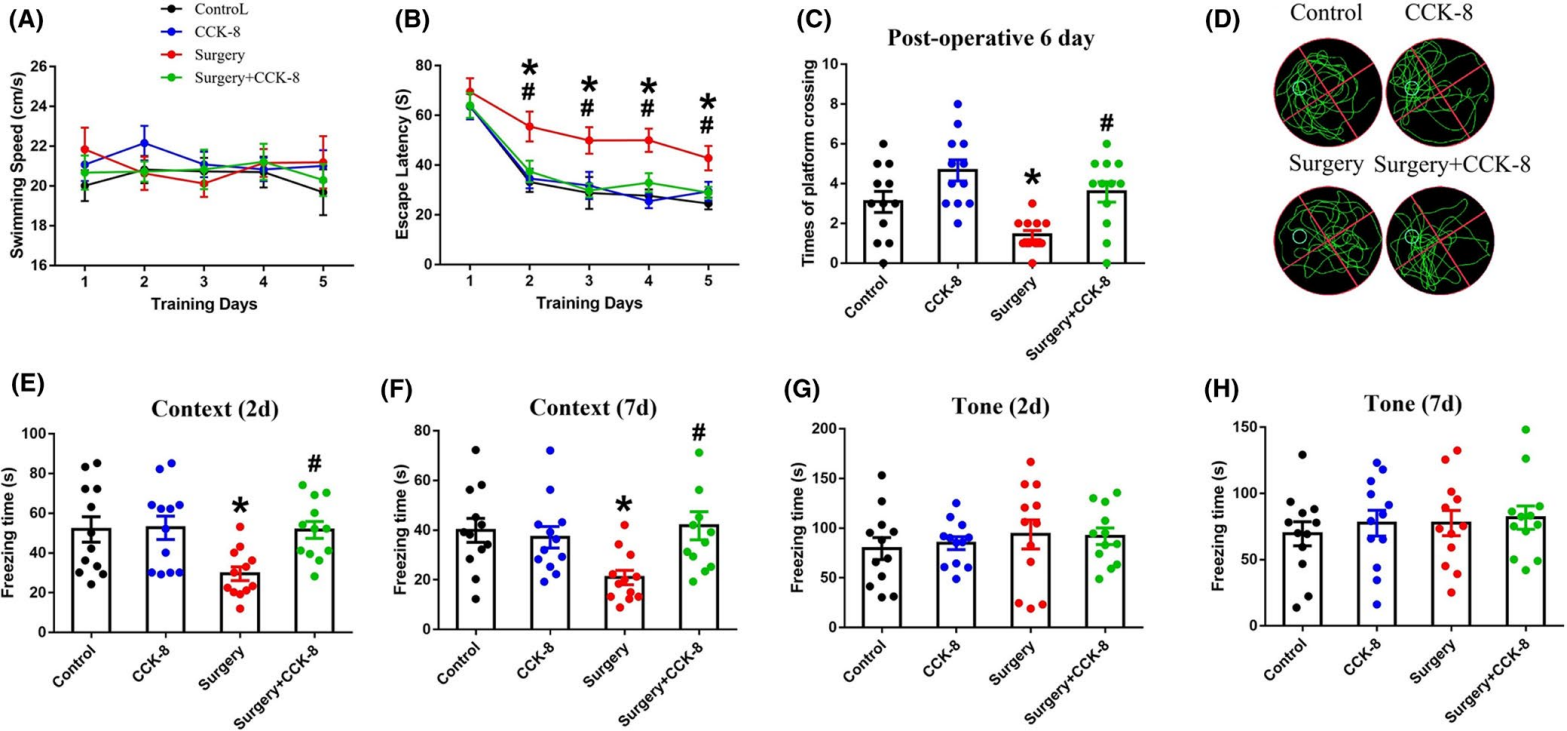
CCK‐8 alleviates hippocampus‐dependent memory impairment in aged dNCR model mice. Swimming speed (A) and escape latency (B) were recorded in the MWM‐place navigation test on days 1–5 after surgery. The number of platform crossings (C, D) was recorded in the MWM‐spatial probe test on day 6 after surgery. Freezing time in the context test (E, F) and tone test (G, H) was recorded on days 2 and 7 after surgery. Data are expressed as the mean ± SEM (repeated measure two‐way ANOVA followed by Bonferroni's post hoc test, *n* = 12 per group).*p < 0.05 versus the control group; *#p* < 0.05 versus the surgery group

We used the MWM to evaluate spatial learning and memory.^31^ There was no significant difference in swimming speed between groups of mice (Figure 2A). However, we found that surgery/anesthesia significantly increased the escape latency on postoperative days 2–5 (Figure 2B) and decreased the number of platform crossings on postoperative day 6 (Figure 2C, D), indicating that the surgery/anesthesia‐induced spatial learning and memory impairment in aged mice. Furthermore, CCK‐8 supplementation decreased escape latency (Figure 2B) and increased the number of platform crossings (Figure 2C, D), indicating that CCK‐8 can alleviate spatial learning and memory impairment in aged dNCR model mice.

The fear conditioning test phase consists of a context test and a tone test. The context test reflects hippocampus‐dependent memory, whereas the tone test reflects hippocampus‐independent memory in rodents.^33^ Surgery/anesthesia significantly decreased the freezing times of aged mice in the context test at 2 and 7 days (Figure 2E, F) postoperatively but not in the tone test (Figure 2G, H), indicating that surgery/anesthesia impaired hippocampus‐dependent memory but not hippocampus‐independent memory, which was consistent with our previous study.^32^ Moreover, CCK‐8 supplementation alleviated hippocampus‐dependent memory impairment at 2 and 7 days postoperatively in aged dNCR model mice (Figure 2E, F). Collectively, the behavioral test results showed that CCK‐8 can relieve impaired cognitive function in aged dNCR model mice.

### 3.3 CCK‐8 upregulates the levels of PSD95 and vGLUT1 in the hippocampus of aged dNCR model mice

**FIGURE 3:**
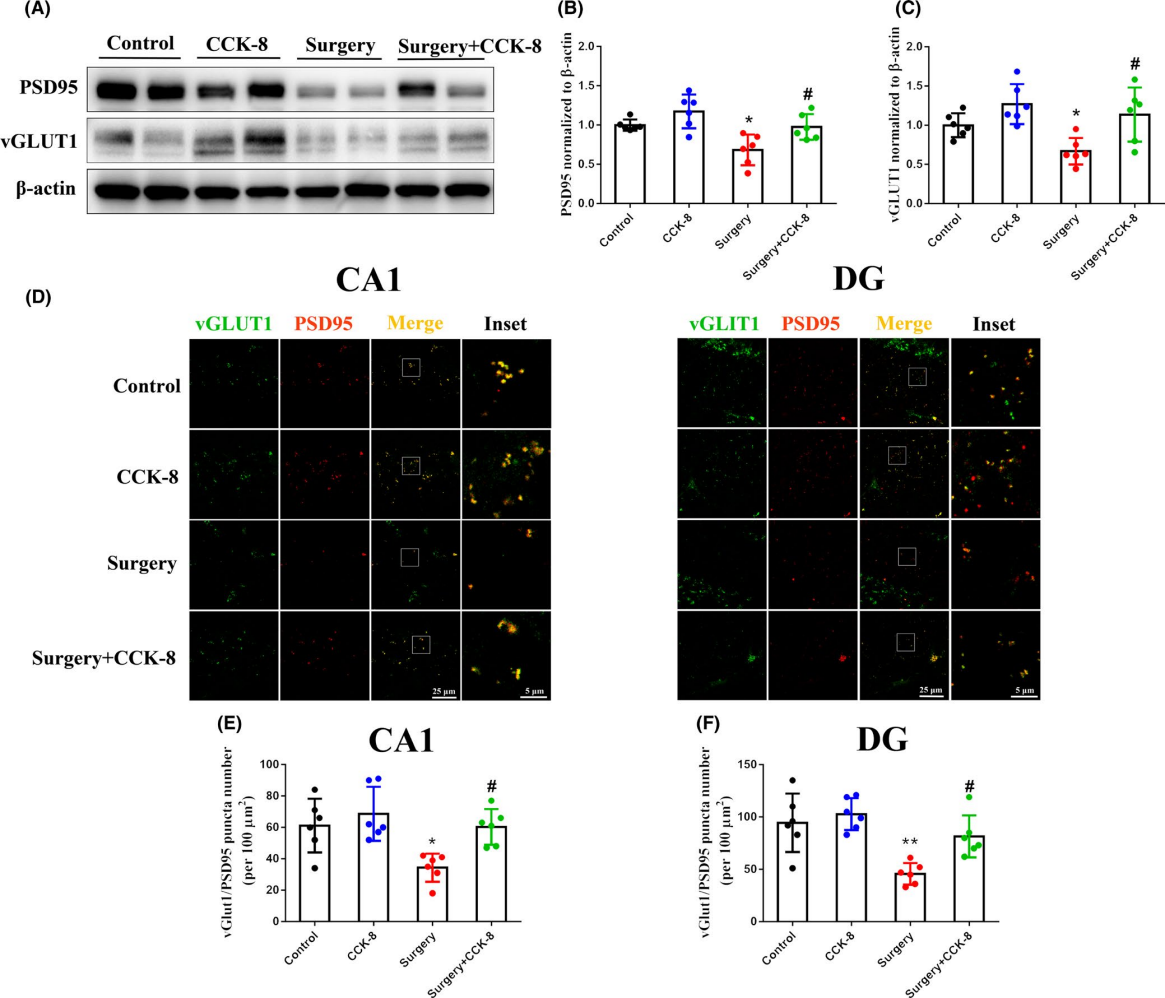
CCK‐8 upregulates the levels of PSD95 and vGLUT1 in the hippocampus of aged dNCR model mice. (A) Western blot detection of PSD95 (A, B) and vGLUT1 (A, C) levels in the hippocampus. Glutamatergic synapses were stained by immunofluorescence (PSD95‐red, vGLUT1‐green, Merged image‐yellow) in hippocampal subregions, including the CA1 (D, E) and DG (D, F). Data are expressed as the mean ± SEM (one‐way ANOVA followed by Bonferroni's post hoc test, *n* = 6 per group).*p < 0.05; **p < 0.01 versus the control group; *#p* < 0.05 versus the surgery group

The density of hippocampal glutamatergic synapses is associated with cognition in rodents and co‐localization of PSD95 and vGLUT1 is commonly used to label the synapses of glutamatergic neurons.^14^ Therefore, we explored the relationship between improvement in cognitive function in response to CCK‐8 and hippocampal glutamatergic synapse density. Surgery/anesthesia significantly decreased the density of glutamatergic synapses, whereas CCK‐8 reversed the reduction in glutamatergic synapse density in hippocampal CA1 and DG regions in aged dNCR model mice (Figure 3). These results indicated that CCK‐8 might improve cognition in aged dNCR model mice by increasing the density of glutamatergic synapses in the hippocampus.

### 3.4 CCK‐8 reduces the density of A1 astrocytes in the hippocampus of aged dNCR model mice

**FIGURE 4:**
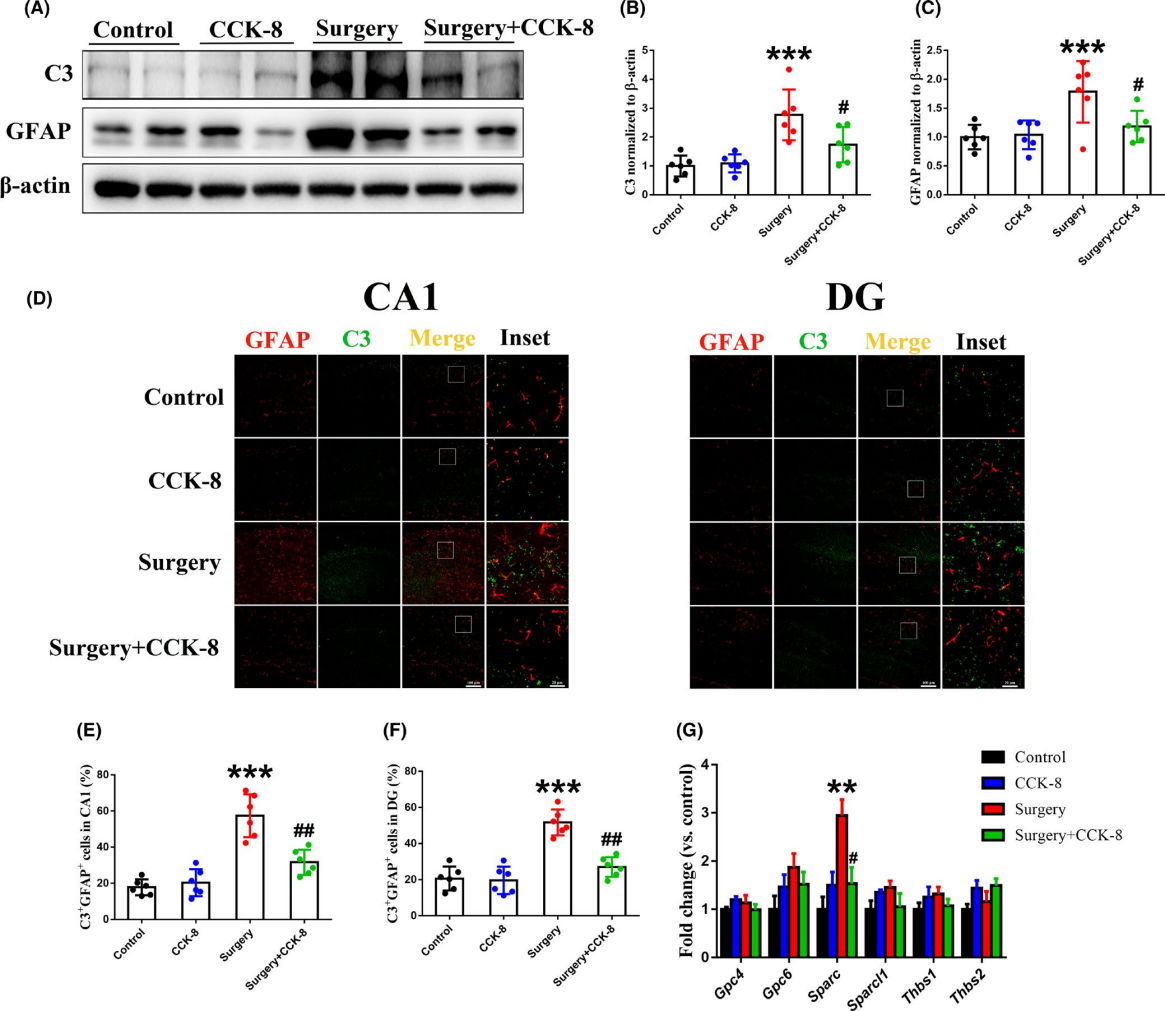
CCK‐8 reduces the density of A1 astrocytes in the hippocampus of aged dNCR model mice. (A) Levels of C3 (A, B) and GFAP (A, C) in the hippocampus were detected by Western blotting. A1 astrocytes were stained by immunofluorescence (GFAP‐red, C3‐green, Merged image‐yellow) in hippocampal subregions, including the CA1 (D, E) and DG (D, F). (G) The levels of *Gpc4*/6, *Sparc*/*Sparcl1*, and *Thbs1*/*Thbs2* expression were tested by qPCR. Data are expressed as the mean ± SEM (one‐way ANOVA followed by Bonferroni's post hoc test, *n* = 6 per group). **p < 0.01; ***p < 0.001 versus the control group; *#p* < 0.05; *##p* < 0.01 versus the surgery group. C3, Complement component 3; Gpc4, Glypican 4; Gpc6, Glypican 6; Sparc, Secreted protein acidic and rich in cysteine; Sparcl1, Sparc‐like 1; Thbs1, Thrombospondin 1; Thbs2, Thrombospondin 2

A1 reactive astrocytes impair the function of synapses^10^; therefore, we investigated whether surgery/anesthesia induces A1 reactive astrocytes and explored the effect of CCK‐8 on A1 reactive astrocytes. As shown in Figure 4A–F, surgery/anesthesia significantly induced A1 reactive astrocytes, as indicated by the specific marker C3,^10^, ^11^ while CCK‐8 decreased the density of A1 reactive astrocytes in the CA1 and DG of the hippocampus. These findings indicated that CCK‐8 might increase the density of glutamatergic synapses by inhibiting the induction of A1 reactive astrocytes.

Astrocytes induce excitatory synapse formation by secreting GPC4/6, SPARC, SPARCL1, and THBS1/2^8^, ^10^; therefore, we investigated the effect of CCK‐8 on the levels of these factors. As can be seen in Figure 4G, surgery/anesthesia increased the expression of *Sparc*, while CCK‐8 reversed this change in the hippocampus of the aged dNCR model mice. SPARC is not synaptogenic, but antagonizes the synaptogenic function of SPARCL1.^5^ Overall, these results indicated that CCK‐8 may decrease the expression of *Sparc* by inhibiting A1 astrocyte activation.

### 3.5 CCK‐8 inhibits the activation of microglia and reduces the levels of TNF‐α, C1q, and IL‐1α in the hippocampus of aged dNCR model mice

**FIGURE 5:**
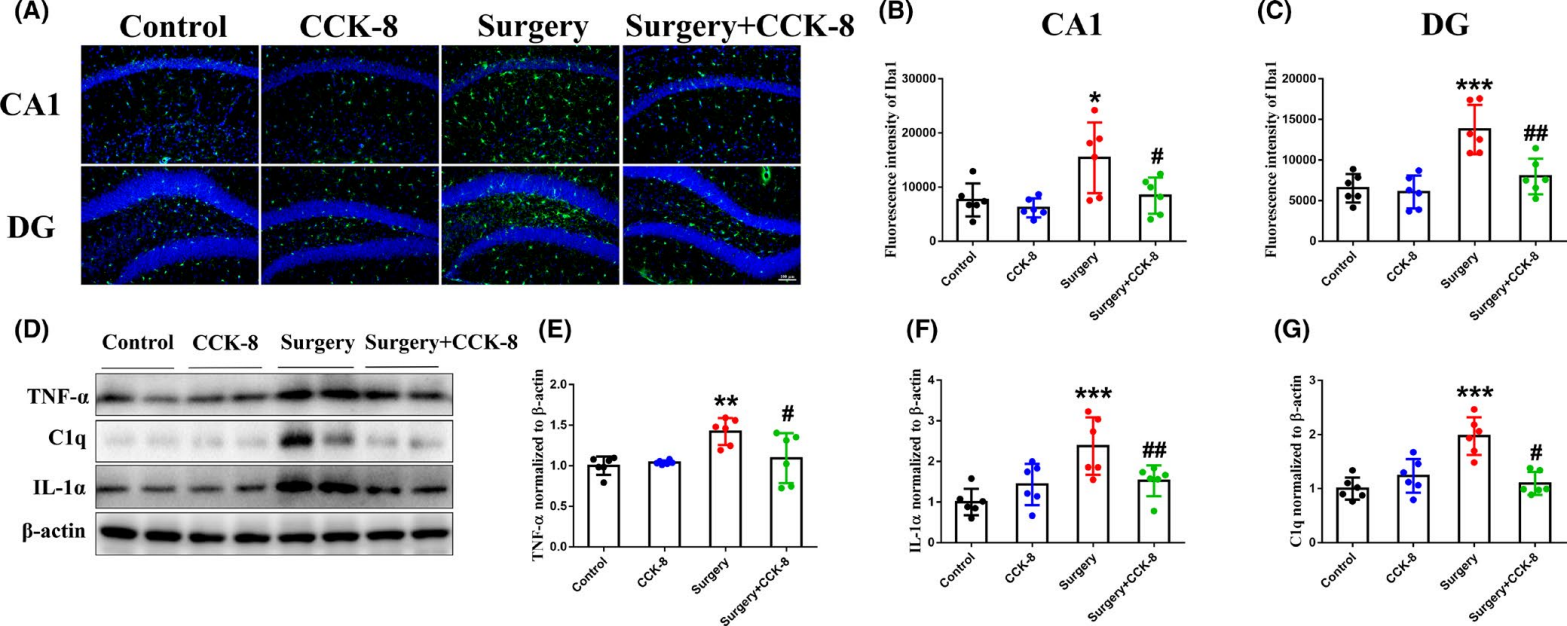
CCK‐8 inhibits the activation of microglia and reduces the levels of TNF‐α, C1q and IL‐1α in the hippocampus of aged dNCR model mice. (A) Activation of microglia in the CA1 (A, B) and DG (A, C) indicated by IBA1 immunofluorescence (green). (D) Western blot detection of TNF‐α (D, E) C1q (D, F) and IL‐1α (D, G) levels in the hippocampus. Data are expressed as the mean ± SEM (one‐way ANOVA followed by Bonferroni's post hoc test, *n* = 6 per group). *p < 0.05; **p < 0.01, ***p < 0.001 versus the control group; *#p* < 0.05, *##p* < 0.01 versus the surgery group

TNF‐α, C1q, and IL‐1α released by activated microglia‐induced A1 reactive astrocytes.^8^ Therefore, we next explored the effect of CCK‐8 on microglia and these factors. CCK‐8 significantly inhibited the activation of microglia (Figure 5A–C) and reduced the levels of TNF‐α, C1q, and IL‐1α (Figure 5D–G) in the hippocampus of aged dNCR model mice. These data indicate that CCK‐8 might reverse A1 astrocyte induction by inhibiting the activation of microglia.

## 4 DISCUSSION

The present study assessed the effect of CCK‐8 on cognitive function in aged dNCR model mice and the potential mechanisms for its action. Surgery/anesthesia can induce cognitive decline in patients and rodents^1^, ^32^; therefore, we used laparotomy under sevoflurane anesthesia to establish a dNCR model in aged mice. We found that surgery/anesthesia decreased the levels of CCK‐8 in the hippocampus of aged mice, especially in the CA1 and DG regions. Moreover, CCK‐8 alleviated impaired cognition and promoted hippocampal glutamatergic synaptogenesis in aged dNCR model mice. Meanwhile, CCK‐8 significantly inhibited microglia activation and induction of A1 reactive astrocytes.

Neuropeptides are small proteins of 3 to approximately 100 amino acids that act as neurotransmitters or neuromodulators to modulate neuronal functions via binding to specific receptors on the surface of cells.^37^ CCK is a gut‐brain peptide that exerts various biological functions in the CNS, including regulation of learning and memory.^38^, ^39^, ^40^ Low levels of CCK are found in AD patient brains and in aged and AD model mice with impaired memory.^22^, ^41^, ^42^, ^43^ Here, we found that surgery/anesthesia‐induced cognitive impairment in aged mice, and significantly decreased the levels of CCK‐8 in the hippocampus, especially in CA1 and DG regions. These results indicated that postoperative cognitive impairment was accompanied by a decrease in CCK‐8 levels in the hippocampus, which is consistent with the above‐mentioned reports on cognitive decline. However, we only detected the concentration of CCK‐8 in the hippocampus 3 days after surgery/anesthesia, which did not reflect the changes of CCK‐8 in the development of dNCR. In future study, we will observe the changes of CCK‐8 in the hippocampus at multiple time points after surgery/anesthesia. Based on these findings, we then explored the effect of CCK‐8 supplementation in aged dNCR model mice. The most obvious finding was that CCK‐8 alleviated the spatial learning and memory impairment of the aged dNCR model mice, indicating that CCK‐8 was an effective therapeutic agent and might be involved in the pathogenesis of dNCR.

Synapses are basic information processing units in the brain.^44^, ^45^ The correct number and type of synaptic connections are essential for normal CNS functions. Specifically, glutamatergic synapses play a pivotal role in memory formation, learning, and emotion.^46^ Xiong and colleagues found that a decreased number of hippocampal glutamatergic synapses induced cognitive impairment in rats with neuropathic pain.^14^ Here, we found that CCK‐8 upregulated the levels of presynaptic vGLUT1 and postsynaptic PSD95, markers of excitatory synapses, in aged dNCR model mice, indicating that CCK‐8 alleviated impaired cognition by promoting glutamatergic synaptogenesis in the hippocampus. Since the regulation of glutamatergic synaptogenesis by CCK have not been reported, ours findings complemented the cognitive protective mechanisms of CCK in dNCR.

In the past 20 years, numerous studies have reported key roles of astrocytes in regulating synapse formation and function.^47^ Inflammation or trauma have been demonstrated to induce A1 reactive astrocytes, which are involved in CNS diseases.^48^ Taylor et al. showed that disruption to immune response networks can induce A1 reactive astrocytes, which participate in the pathogenesis of AD‐related dementia, such as early‐stage cerebral amyloid angiopathy.^49^ Furthermore, etomidate induces synaptic inhibition and cognitive decline in aged mice by inducing A1 reactive astrocytes.^12^ Here, we found that CCK‐8 significantly inhibited A1 reactive astrocyte induction in the hippocampus. These results are in agreement with those of Asrican et al. They showed that CCK released from CCK interneurons promoted neurogenic proliferation of radial glia‐like neural stem cells by inhibiting A1 reactive astrocytes.^25^ Overall, our findings indicated that CCK‐8 upregulates the density of glutamatergic synapses by inhibiting A1 reactive astrocyte induction. However, the relationship between A1 reactive astrocytes and glutamatergic synapses remains unclear.

Culture of retinal ganglion cells with A1 astrocytes resulted in 50% fewer synapses compared with culture with normal astrocytes, which indicates a supportive property of astrocytes in synaptogenesis.^10^ Astrocytes regulate synaptogenesis by secreting several factors, such as THBS1/2, GPC4/6 and SPARC/SPARCL1.^5^, ^50^, ^51^ SPARC and SPARCL1 are members of the Sparc family.^52^ SPARCL1 is located on excitatory synapses in the CNS and induces the formation of synapses between cultured retinal ganglion cells.^5^, ^53^ Although SPARC cannot directly modulate synaptogenesis, it can specifically antagonize the synapse formation function of SPARCL1.^5^ Therefore, we determined the mRNA levels of the above‐mentioned factors in the hippocampus, and speculated whether neurotoxic A1 astrocytes damaged hippocampal glutamatergic synaptogenesis by modulating the expression of these factors in aged mice. The most interesting finding was that surgery/anesthesia only increased the levels of *Sparc* mRNA, while CCK‐8 reversed this increase. Asrican et al. found that decreased *Gpc6* and *Sparcl1* expression in A1 reactive astrocytes was accompanied by increased expression of *Thbs1*/*2*,^10^ which was not consistent with our findings. A possible explanation for this might be that Asrican et al. generated A1 reactive astrocytes *in vitro* by stimulating them with IL‐1α, TNF‐α, and C1q, while we detected the expression of *Thbs1*/*2*, *Gpc4*/*6* and *Sparc*/*Sparcl1 in vivo*. Ours finding demonstrated that A1 reactive astrocytes might damage glutamatergic synapses via increasing *Sparc* in dNCR, which is a complement to the mechanism of neuronal damage by A1 reactive astrocytes. Nevertheless, the mechanism by which CCK‐8 inhibits *Sparc* expression in the aged dNCR model mice warrants further investigation.

A1 reactive astrocytes are mainly induced by IL‐1α, TNF‐α, and C1q, which are predominately released by activated microglia.^10^, ^54^ CCK‐8 alleviates methamphetamine‐induced microglial activation via CCK2 receptors^55^; therefore, we investigated the effect of CCK‐8 on microglia. We showed that CCK‐8 significantly inhibits activation of microglia and the release of inflammatory factors, including IL‐1α, TNF‐α and C1q. To the best of our knowledge, this is the first report of CCK‐8 to inhibit the release of IL‐1α, TNF‐α, and C1q, which complements the neuroprotective mechanism of CCK‐8. Li and colleagues found that microglia depletion before etomidate administration could inhibit etomidate‐induced A1 reactive astrocyte activation.^12^ Furthermore, a rat model of chronic postsurgical pain confirmed that microglia induce A1 reactive astrocytes via the CXCR7/PI3K/Akt pathway.^11^ Thus, we speculated that CCK‐8 reduced A1 reactive astrocyte induction by inhibiting microglia activation. However, these data must be interpreted with caution because CCK2 receptors are also located on astrocytes.^25^ CCK released by CCK interneurons increases neurogenic proliferation of glia‐like neural stem cells by inhibiting A1 reactive astrocytes.^25^ We did not use astrocyte CCK2 receptor conditional knockout mice to verify the effect of CCK on neurogenic proliferation; therefore, the direct effect of CCK on astrocytes via CCK2 receptors cannot be excluded. In summary, CCK‐8 might inhibit A1 reactive astrocyte induction by reducing the levels of IL‐1α, TNF‐α, and C1q.

The present study had several limitations. First, we only used female mice, although a recent study demonstrated no difference in behavior and physiological state between female and male mice.^56^ Second, the lidocaine cream applied relieves incisional pain, but not visceralgia in aged mice. Therefore, we cannot exclude the effect of postoperative visceralgia on the cognition of mice. Third, we did not assess surgery/anesthesia‐induced peripheral inflammatory factors entering the CNS via a damaged blood brain barrier, which is one of the most important pathogenic mechanisms of dNCR.^57^, ^58^ Additionally, CCK‐8 can reduce the release of peripheral inflammatory factors via vagal afferent CCK1 receptors^23^; therefore, peripheral anti‐inflammation effects of CCK‐8 may play a role in dNCR. Fourth, long‐term potentiation plays a pivotal role in synaptic transmission plasticity, which is often used in studies of learning and memory.^59^ However, we did not observed the effect of CCK‐8 on long‐term potentiation. Finally, we focused only on short‐term postoperative cognitive impairment; however, the effects of CCK‐8 on long‐term cognition in aged mice warrant further study.

## 5 CONCLUSIONS

CCK‐8 alleviates postoperative cognitive impairment and promotes glutamatergic synaptogenesis in the hippocampus via inhibition of A1 reactive astrocytes, which may be mediated by inhibition of microglia activation. These findings indicate CCK‐8 to be a potential therapeutic target for dNCR.

## CONFLICT OF INTEREST

The authors declare that there is no conflict of interest.

## Supporting information

Supplementary Material

## Data Availability

The datasets analyzed in the present study are available from the corresponding author on reasonable request.

## References

[1] Deiner S , Silverstein JH . Postoperative delirium and cognitive dysfunction. Br J Anaesth. 2009;103:i41‐i46. 20007989 10.1093/bja/aep291 PMC2791855

[2] Newman S , Stygall J , Hirani S , Shaefi S , Maze M . Postoperative cognitive dysfunction after noncardiac surgery: a systematic review. Anesthesiology. 2007;106(3):572‐590. 17325517 10.1097/00000542-200703000-00023

[3] Steinmetz J , Christensen KB , Lund T , Lohse N , Rasmussen LS . Long‐term consequences of postoperative cognitive dysfunction. Anesthesiology. 2009;110(3):548‐555. 19225398 10.1097/ALN.0b013e318195b569

[4] Linnerbauer M , Wheeler MA , Quintana FJ . Astrocyte crosstalk in CNS inflammation. Neuron. 2020;108(4):608‐622. 32898475 10.1016/j.neuron.2020.08.012 PMC7704785

[5] Kucukdereli H , Allen NJ , Lee AT , et al. Control of excitatory CNS synaptogenesis by astrocyte‐secreted proteins Hevin and SPARC. Proc Natl Acad Sci USA. 2011;108(32):E440‐449. 21788491 10.1073/pnas.1104977108 PMC3156217

[6] Femenía T , Giménez‐Cassina A , Codeluppi S . Disrupted neuroglial metabolic coupling after peripheral surgery. J Neurosci. 2018;38(2):452‐464. 29175959 10.1523/JNEUROSCI.1797-17.2017 PMC5761619

[7] Liddelow S , Barres B . SnapShot: Astrocytes in health and disease. Cell. 2015;162(5):1170.e1171. 26317476 10.1016/j.cell.2015.08.029

[8] Liddelow SA , Barres BA . Reactive astrocytes: production, function, and therapeutic potential. Immunity. 2017;46(6):957‐967. 28636962 10.1016/j.immuni.2017.06.006

[9] Terrando N , Gómez‐Galán M , Yang T , et al. Aspirin‐triggered resolvin D1 prevents surgery‐induced cognitive decline. FASEB J. 2013;27(9):3564‐3571. 23709617 10.1096/fj.13-230276

[10] Liddelow SA , Guttenplan KA , Clarke LE , et al. Neurotoxic reactive astrocytes are induced by activated microglia. Nature. 2017;541(7638):481‐487. 28099414 10.1038/nature21029 PMC5404890

[11] Li T , Liu T , Chen X , et al. Microglia induce the transformation of A1/A2 reactive astrocytes via the CXCR7/PI3K/Akt pathway in chronic post‐surgical pain. J Neuroinflammation. 2020;17(1):211. 32665021 10.1186/s12974-020-01891-5 PMC7362409

[12] Li D , Chen M , Meng T , Fei J . Hippocampal microglial activation triggers a neurotoxic‐specific astrocyte response and mediates etomidate‐induced long‐term synaptic inhibition. J Neuroinflammation. 2020;17(1):109. 32264970 10.1186/s12974-020-01799-0 PMC7140340

[13] Nguyen AQ , Sutley S , Koeppen J , Mina K , Woodruff S , Hanna S . Astrocytic ephrin‐B1 controls excitatory‐inhibitory balance in developing hippocampus. J Neurosci. 2020;40(36):6854‐6871. 32801156 10.1523/JNEUROSCI.0413-20.2020 PMC7470912

[14] Xiong B , Zhang W , Zhang L , et al. Hippocampal glutamatergic synapses impairment mediated novel‐object recognition dysfunction in rats with neuropathic pain. Pain. 2020;161(8):1824‐1836. 32701842 10.1097/j.pain.0000000000001878

[15] Sadeghi M , Radahmadi M , Reisi P . Effects of repeated treatment with cholecystokinin sulfated octapeptide on passive avoidance memory under chronic restraint stress in male rats. Advanced Biomedical Research. 2015;4:150. 26380235 10.4103/2277-9175.161577 PMC4550951

[16] Schiffmann SN , Teugels E , Halleux P , Menu R , Vanderhaeghen JJ . Cholecystokinin mRNA detection in rat spinal cord motoneurons but not in dorsal root ganglia neurons. Neurosci Lett. 1991;123(1):123‐126. 2062448 10.1016/0304-3940(91)90173-q

[17] Yang S , Wen D , Dong M , et al. Effects of cholecystokinin‐8 on morphine‐induced spatial reference memory impairment in mice. Behav Brain Res. 2013;256:346‐353. 23994230 10.1016/j.bbr.2013.08.033

[18] Petrella C , Di Certo MG , Barbato C , et al. Neuropeptides in Alzheimer's disease: an update. Curr Alzheimer Res. 2019;16(6):544‐558. 31456515 10.2174/1567205016666190503152555

[19] Ballaz S , Espinosa N , Bourin M . Does endogenous cholecystokinin modulate alcohol intake? Neuropharmacology. 2021;193:108539. 33794246 10.1016/j.neuropharm.2021.108539

[20] Rehfeld JF . Cholecystokinin and the hormone concept. Endocr Conn. 2021;10(3):R139‐r150. 10.1530/EC-21-0025 PMC8052576 33640870

[21] Zanoveli JM , Netto CF , Guimarães FS , Zangrossi H Jr . Systemic and intra‐dorsal periaqueductal gray injections of cholecystokinin sulfated octapeptide (CCK‐8s) induce a panic‐like response in rats submitted to the elevated T‐maze. Peptides. 2004;25(11):1935‐1941. 15501525 10.1016/j.peptides.2004.06.016

[22] Croll SD , Chesnutt CR , Greene NA , Lindsay RM , Wiegand SJ . Peptide immunoreactivity in aged rat cortex and hippocampus as a function of memory and BDNF infusion. Pharmacol Biochem Behav. 1999;64(3):625‐635. 10548280 10.1016/s0091-3057(99)00122-7

[23] Plagman A , Hoscheidt S , McLimans KE , et al. Cholecystokinin and Alzheimer's disease: a biomarker of metabolic function, neural integrity, and cognitive performance. Neurobiol Aging. 2019;76:201‐207. 30739077 10.1016/j.neurobiolaging.2019.01.002 PMC6425756

[24] Choi JG , Jeong M , Joo BR , et al. Reduced Levels of Intestinal Neuropeptides and Neurotrophins in Neurotoxin‐Induced Parkinson Disease Mouse Models. J Neuropathol Exp Neurol. 2021;80(1):15‐20. 33000126 10.1093/jnen/nlaa113

[25] Asrican B , Wooten J , Li YD , et al. Neuropeptides modulate local astrocytes to regulate adult hippocampal neural stem cells. Neuron. 2020;108(2):349‐366.e346. 32877641 10.1016/j.neuron.2020.07.039 PMC7606593

[26] Percie du Sert N , Hurst V , Ahluwalia A , et al. The ARRIVE guidelines 2.0: Updated guidelines for reporting animal research. J Cereb Blood Flow Metab. 2020;40(9):1769‐1777. 32663096 10.1177/0271678X20943823 PMC7430098

[27] Mi X , Cao Y , Li Y , et al. The non‐peptide angiotensin‐(1–7) Mimic AVE 0991 attenuates delayed neurocognitive recovery after laparotomy by reducing neuroinflammation and restoring blood‐brain barrier integrity in aged rats. Front Aging Neurosci. 2021;13:624387. 33658918 10.3389/fnagi.2021.624387 PMC7917118

[28] Jia X , Zhang L , Zhang W , et al. Melatonin ameliorates the sleep disorder induced by surgery under sevoflurane anaesthesia in aged mice. Basic Clin Pharmacol Toxicol. 2021;128(2):256‐267. 32975883 10.1111/bcpt.13498

[29] Zhang W , Xiong BR , Zhang LQ , et al. Disruption of the GABAergic system contributes to the development of perioperative neurocognitive disorders after anesthesia and surgery in aged mice. CNS Neurosci Ther. 2020;26(9):913‐924. 32488976 10.1111/cns.13388 PMC7415208

[30] Voits M , Hasenöhrl RU , Huston JP , Fink H . Repeated treatment with cholecystokinin octapeptide improves maze performance in aged Fischer 344 rats. Peptides. 2001;22(8):1325‐1330. 11457528 10.1016/s0196-9781(01)00459-4

[31] Vorhees CV , Williams MT . Morris water maze: procedures for assessing spatial and related forms of learning and memory. Nat Protoc. 2006;1(2):848‐858. 17406317 10.1038/nprot.2006.116 PMC2895266

[32] Chen L , Dong R , Lu YY , et al. MicroRNA‐146a protects against cognitive decline induced by surgical trauma by suppressing hippocampal neuroinflammation in mice. Brain Behav Immun. 2019;78:188‐201. 30685530 10.1016/j.bbi.2019.01.020

[33] Vizcaychipi MP , Xu L , Barreto GE , Ma D , Maze M , Giffard RG . Heat shock protein 72 overexpression prevents early postoperative memory decline after orthopedic surgery under general anesthesia in mice. Anesthesiology. 2011;114(4):891‐900. 21317632 10.1097/ALN.0b013e31820ad3ce PMC3063324

[34] Ye JS , Chen L , Lu YY , Lei SQ , Peng M , Xia ZY . SIRT3 activator honokiol ameliorates surgery/anesthesia‐induced cognitive decline in mice through anti‐oxidative stress and anti‐inflammatory in hippocampus. CNS Neurosci Ther. 2019;25(3):355‐366. 30296006 10.1111/cns.13053 PMC6488903

[35] Faul F , Erdfelder E , Lang AG , Buchner A . G*Power 3: a flexible statistical power analysis program for the social, behavioral, and biomedical sciences. Behav Res Methods. 2007;39(2):175‐191. 17695343 10.3758/bf03193146

[36] Sun L , Dong R , Xu X , Yang X , Peng M . Activation of cannabinoid receptor type 2 attenuates surgery‐induced cognitive impairment in mice through anti‐inflammatory activity. J Neuroinflamm. 2017;14(1):138. 10.1186/s12974-017-0913-7 PMC5518095 28724382

[37] Hallberg M . Neuropeptides: metabolism to bioactive fragments and the pharmacology of their receptors. Med Res Rev. 2015;35(3):464‐519. 24894913 10.1002/med.21323

[38] Flood JF , Smith GE , Morley JE . Modulation of memory processing by cholecystokinin: dependence on the vagus nerve. Science. 1987;236(4803):832‐834. 3576201 10.1126/science.3576201

[39] Schneider R , Osterburg J , Buchner A , Pietrowsky R . Effect of intranasally administered cholecystokinin on encoding of controlled and automatic memory processes. Psychopharmacology. 2009;202(4):559‐567. 18836704 10.1007/s00213-008-1332-3

[40] Chen X , Li X , Wong YT , et al. Cholecystokinin release triggered by NMDA receptors produces LTP and sound‐sound associative memory. Proc Natl Acad Sci USA. 2019;116(13):6397‐6406. 30850520 10.1073/pnas.1816833116 PMC6442640

[41] Löfberg C , Harro J , Gottfries CG , Oreland L . Cholecystokinin peptides and receptor binding in Alzheimer's disease. J Neural Transm. 1996;103(7):851‐860. 8872869 10.1007/BF01273363

[42] Perry RH , Dockray GJ , Dimaline R , Perry EK , Blessed G , Tomlinson BE . Neuropeptides in Alzheimer's disease, depression and schizophrenia. A post mortem analysis of vasoactive intestinal peptide and cholecystokinin in cerebral cortex. J Neurol Sci. 1981;51(3):465‐472. 6268760 10.1016/0022-510x(81)90123-4

[43] Diez M , Danner S , Frey P , et al. Neuropeptide alterations in the hippocampal formation and cortex of transgenic mice overexpressing beta‐amyloid precursor protein (APP) with the Swedish double mutation (APP23). Neurobiol Dis. 2003;14(3):579‐594. 14678773 10.1016/j.nbd.2003.08.003

[44] Mayford M , Siegelbaum SA , Kandel ER . Synapses and memory storage. Cold Spring Harb Perspect Biol. 2012;4(6):a005751. 22496389 10.1101/cshperspect.a005751 PMC3367555

[45] Südhof TC , Malenka RC . Understanding synapses: past, present, and future. Neuron. 2008;60(3):469‐476. 18995821 10.1016/j.neuron.2008.10.011 PMC3243741

[46] Hayashi T . Post‐translational palmitoylation of ionotropic glutamate receptors in excitatory synaptic functions. Br J Pharmacol. 2021;178(4):784‐797. 32159240 10.1111/bph.15050

[47] Chung WS , Allen NJ , Eroglu C . Astrocytes control synapse formation, function, and elimination. Cold Spring Harb Perspect Biol. 2015;7(9):a020370. 25663667 10.1101/cshperspect.a020370 PMC4527946

[48] Zamanian JL , Xu L , Foo LC , et al. Genomic analysis of reactive astrogliosis. J Neurosci. 2012;32(18):6391‐6410. 22553043 10.1523/JNEUROSCI.6221-11.2012 PMC3480225

[49] Taylor X , Cisternas P , You Y , et al. A1 reactive astrocytes and a loss of TREM2 are associated with an early stage of pathology in a mouse model of cerebral amyloid angiopathy. J Neuroinflamm. 2020;17(1):223. 10.1186/s12974-020-01900-7 PMC7382050 32711525

[50] Christopherson KS , Ullian EM , Stokes CC , et al. Thrombospondins are astrocyte‐secreted proteins that promote CNS synaptogenesis. Cell. 2005;120(3):421‐433. 15707899 10.1016/j.cell.2004.12.020

[51] Allen NJ , Bennett ML , Foo LC , et al. Astrocyte glypicans 4 and 6 promote formation of excitatory synapses via GluA1 AMPA receptors. Nature. 2012;486(7403):410‐414. 22722203 10.1038/nature11059 PMC3383085

[52] Brekken RA , Sage EH . SPARC, a matricellular protein: at the crossroads of cell‐matrix communication. Matrix Biol. 2001;19(8):816‐827. 11223341 10.1016/s0945-053x(00)00133-5

[53] Lively S , Ringuette MJ , Brown IR . Localization of the extracellular matrix protein SC1 to synapses in the adult rat brain. Neurochem Res. 2007;32(1):65‐71. 17151913 10.1007/s11064-006-9226-4

[54] Sterling JK , Adetunji MO , Guttha S , et al. GLP‐1 receptor agonist NLY01 reduces retinal inflammation and neuron death secondary to ocular hypertension. Cell Rep. 2020;33(5):108271. 33147455 10.1016/j.celrep.2020.108271 PMC7660987

[55] Gou H , Sun D , Hao L , et al. Cholecystokinin‐8 attenuates methamphetamine‐induced inflammatory activation of microglial cells through CCK2 receptor. Neurotoxicology. 2020;81:70‐79. 32916201 10.1016/j.neuro.2020.09.001

[56] Shansky RM . Are hormones a "female problem" for animal research? Science. 2019;364(6443):825‐826. 31147505 10.1126/science.aaw7570

[57] Li Z , Mo N , Li L , et al. Surgery‐induced hippocampal angiotensin II elevation causes blood‐brain barrier disruption via MMP/TIMP in aged rats. Front Cell Neurosci. 2016;10:105. 27199659 10.3389/fncel.2016.00105 PMC4844612

[58] Subramaniyan S , Terrando N . Neuroinflammation and perioperative neurocognitive disorders. Anesth Analg. 2019;128(4):781‐788. 30883423 10.1213/ANE.0000000000004053 PMC6437083

[59] Díaz‐Alonso J , Nicoll RA . AMPA receptor trafficking and LTP: Carboxy‐termini, amino‐termini and TARPs. Neuropharmacology. 2021;197:108710. 34271016 10.1016/j.neuropharm.2021.108710 PMC9122021

