# Supplementary material for: Cholecystokinin octapeptide improves hippocampal glutamatergic synaptogenesis and postoperative cognition by inhibiting induction of A1 reactive astrocytes in aged mice
Source: CNS Neurosci Ther. 2021 Aug 17;27(11):1374–84. doi: 10.1111/cns.13718 (PMC8504528; doi:10.1111/cns.13718)

Full unedited blot for Figure 3

PSD95-Full unedited blot for Figure 3

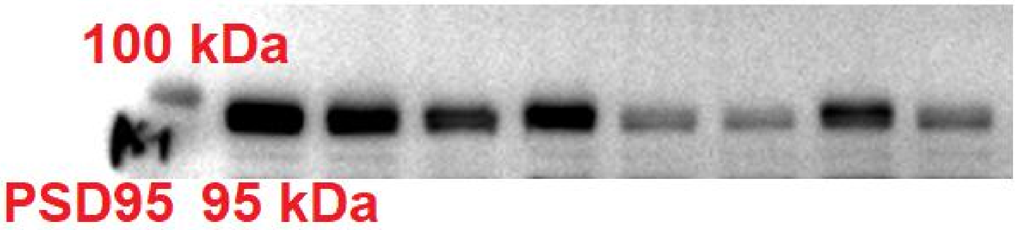

vGLUT1-Full unedited blot for Figure 3

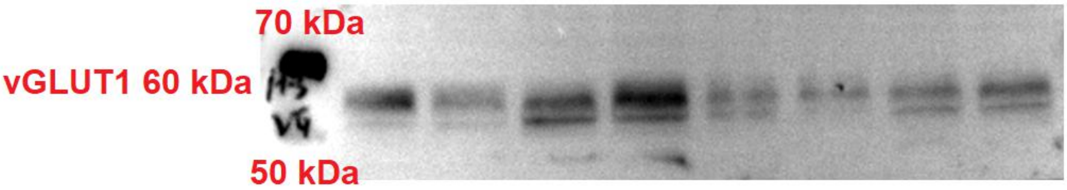

β-actin-Full unedited blot for Figure 3

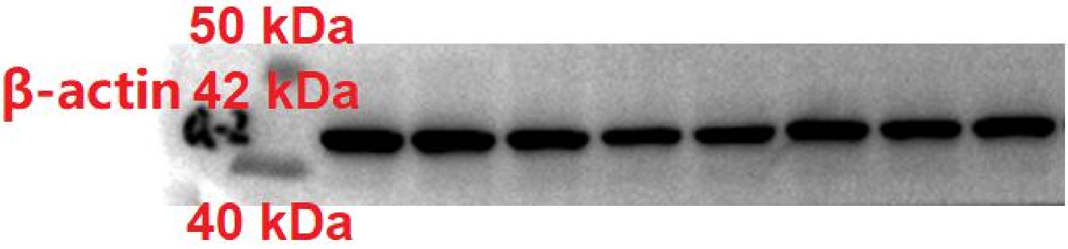

Full unedited blot for Figure 4

C3-Full unedited blot for Figure 4

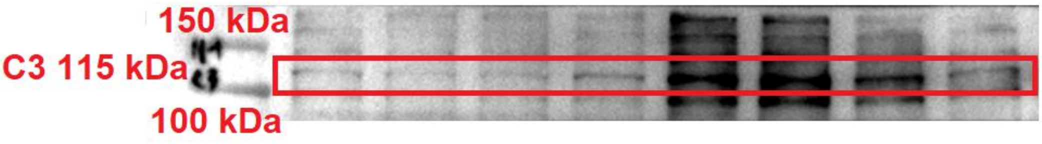

GFAP-Full unedited blot for Figure 4

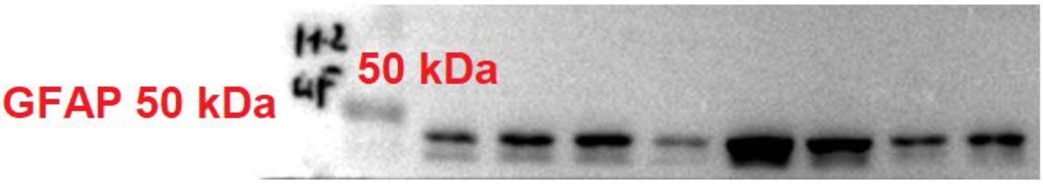

β-actin-Full unedited blot for Figure 4

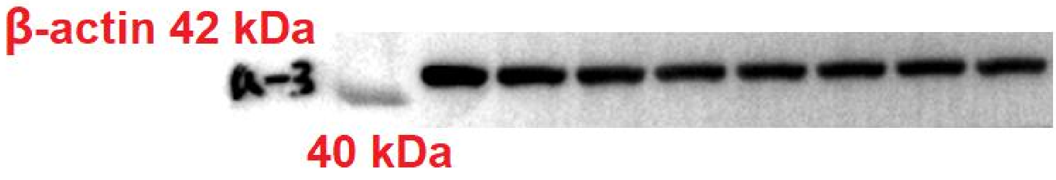

Full unedited blot for Figure 5

TNF-α-Full unedited blot for Figure 5

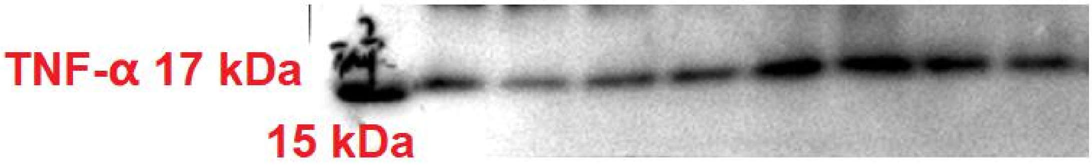

C1q-Full unedited blot for Figure 5

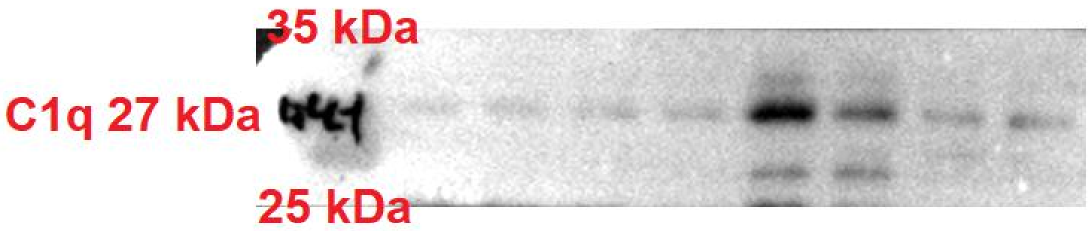

IL-1α-Full unedited blot for Figure 5

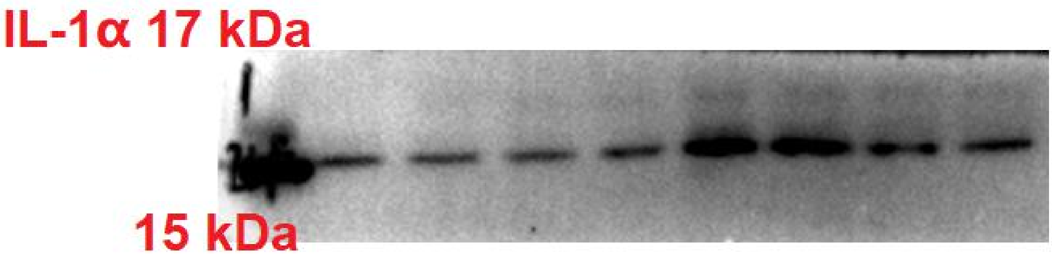

β-actin-Full unedited blot for Figure 5

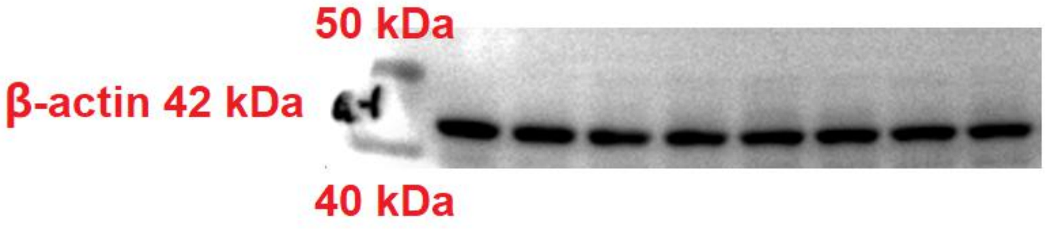

Supplement: Supplementary file 1 — Supplementary Material [file CNS-27-1374-s001.pdf]
